# Tailoring Inflammatory Biomarker Assessment in Axial Spondyloarthritis: A Comparative Study of Erythrocyte Sedimentation Rate and C-Reactive Protein Across Disease Profiles

**DOI:** 10.3390/jpm15080329

**Published:** 2025-07-25

**Authors:** Rubén Queiro, Sara Alonso, Stefanie Burger, Estefanía Pardo, Ignacio Braña, Marta Loredo, Mercedes Alperi

**Affiliations:** 1 Rheumatology Division, Hospital Universitario Central de Asturias, 33011 Oviedo, Spain; saraalonsocastro@hotmail.com (S.A.); stefanie.nam@gmail.com (S.B.); estefaniapardoc@gmail.com (E.P.); i.brana.abascal@hotmail.es (I.B.); mloredomart@gmail.com (M.L.); mercedes_alperi@hotmail.com (M.A.); 2 Department of Medicine, Oviedo University School of Medicine, 33011 Oviedo, Spain; 3 Translational Immunology Division, Health Research Institute of the Principality of Asturias (ISPA), 33011 Oviedo, Spain

**Keywords:** axial spondyloarthritis, biomarkers, disease burden, disease impact

## Abstract

**Background**: Personalized medicine in axial spondyloarthritis (axSpA) requires accurate tools to assess inflammation and tailor disease monitoring. The role of traditional biomarkers such as erythrocyte sedimentation rate (ESR) and C-reactive protein (CRP) remains controversial due to limited sensitivity and variability across disease profiles. **Objective**: To compare the performance of ESR and CRP in different clinical scenarios of axSpA, including disease activity, functional impact, severity, disease duration, and exposure to biologic therapy. **Methods:** We conducted a cross-sectional analysis of 330 patients with axSpA. Correlations among ESR, CRP, and composite disease indices were evaluated. The discriminatory capacity of each biomarker for relevant clinical thresholds was analyzed using ROC curves and optimal cut-offs identified by the Youden index. **Results**: ESR showed broader correlations with disease impact and activity scores than CRP. While both markers had low sensitivity overall, they were highly specific for identifying patients with very high disease activity in select scenarios. ESR ≥ 8.5 mm/h and CRP ≥ 1.88 mg/dL were strongly discriminatory in patients not exposed to biologics. CRP ≥ 0.56 mg/dL showed good performance in early disease. **Conclusions**: Both ESR and CRP provide complementary insights into disease activity in axSpA. ESR may offer a broader reflection of disease burden beyond inflammation. These results support a more personalized biomarker strategy in real-world axSpA management, adapted to patient profile and treatment context.

## 1. Introduction

Axial spondyloarthritis (axSpA) is a chronic inflammatory disease primarily affecting the spine and sacroiliac joints, often associated with peripheral arthritis, uveitis, and inflammatory bowel disease. The global prevalence of axSpA is increasing, posing significant challenges in terms of early diagnosis and appropriate management [1]. This condition primarily affects young adults in their productive years, underscoring the importance of early diagnosis and effective treatment to prevent disease progression and improve patient quality of life. However, due to the clinical heterogeneity of the disease, accurately assessing disease activity and its impact remains challenging [1].

Accurate evaluation of disease activity is crucial for personalized treatment strategies in axSpA. While patient-reported indices such as the Bath Ankylosing Spondylitis Disease Activity Index (BASDAI) provide valuable insight into symptom burden, composite indices like the Ankylosing Spondylitis Disease Activity Score (ASDAS)—which incorporate inflammatory biomarkers—have become central tools for clinical decision making [1,2,3].

In this context, inflammatory biomarkers such as erythrocyte sedimentation rate (ESR) and C-reactive protein (CRP) have long been used as assessment tools to monitor disease activity in axSpA. These biomarkers reflect the presence of systemic inflammation, but their sensitivity and specificity in the context of axSpA are limited, generating debate over their utility in guiding treatment decisions effectively. It is estimated that up to 50% of patients with clinically active axSpA present with normal levels of these biomarkers. This variability challenges their utility in a “one-size-fits-all” approach and underlines the need for more nuanced, individualized interpretation of biomarker data in clinical practice [4,5].

In recent years, there has been a growing effort to integrate personalized medicine approaches into the management of axSpA. Personalized medicine is based on the concept that treatments should be tailored not only to the general characteristics of the disease but also to the specific traits of each patient, including their genetic, phenotypic, and therapeutic factors. Identifying more specific and sensitive biomarkers for disease monitoring is crucial to this approach. Thus, biomarker analysis must consider not only performance in terms of sensitivity and specificity but also the ability to reflect functional impact and the overall disease burden, particularly in the context of different disease stages and exposure to biological therapies.

This study aims to compare the performance of traditional biomarkers, ESR and CRP, across various clinical scenarios of patients with axSpA, including disease activity, functional impact, severity, disease duration, and exposure to biologic therapies. Through a cross-sectional analysis of more than 300 patients, we evaluate the correlations between these biomarkers and disease activity indices and analyze their discriminatory capacity to identify relevant clinical thresholds. Based on these findings, we aim to promote a more personalized interpretation of biomarkers in clinical practice, tailored to each patient’s specific profile, which could contribute to improving therapeutic decision making and the management of axSpA.

## 2. Materials and Methods

### 2.1. Study Population and Ethical Issues

This cross-sectional study was conducted at a single academic center and included 330 adult patients who fulfilled the ASAS classification criteria for axial spondyloarthritis (axSpA) [6]. Participants were enrolled from a dedicated rheumatology unit with expertise in the diagnosis and management of axSpA between January and October 2023. All individuals provided written informed consent prior to inclusion. Given the retrospective nature of data collection, formal approval by an ethics committee was not required. The study adhered to Spanish legislation (Order SAS/3470/2009 from the Ministry of Health and Social Policy), relevant institutional and national guidelines, and the ethical principles of the Declaration of Helsinki. Compliance with the European General Data Protection Regulation (GDPR, Regulation EU 2016/679) was also maintained throughout the research process.

### 2.2. Clinical and Laboratory Assessments

Sociodemographic, clinical, analytical, and radiographic variables were collected, as well as those related to the therapies received. For the purposes of the study, the following composite indices were included: BASDAI, BASFI (Bath Ankylosing Spondylitis Functional Index), CRP-based ASDAS (CRP-ASDAS), ESR-based ASDAS (ESR-ASDAS), ASAS-HI (ASAS Health Index), and RAPID3 (Routine Assessment of Patient Index Data 3). Axial structural damage was evaluated by means of the mSASSS (modified Stoke Ankylosing Spondylitis Spinal Score). ESR and CRP levels were extracted from the clinical record, ensuring that they corresponded to visits when clinical indices were also assessed. Cases with evidence of concurrent infection or unrelated inflammatory conditions were excluded. According to international standards, we have set the normal value for ESR at a value less than or equal to 20 mm/h in men and a value less than or equal to 30 mm/h in women. The normal limit for CRP was estimated at a value less than 0.5 mg/dL according to our laboratory standards.

### 2.3. Outcome Composite Indices

BASDAI: The BASDAI is a widely used measure of disease activity in patients with axSpA, specifically ankylosing spondylitis (AS). It comprises six questions evaluating the severity of fatigue, spinal pain, joint pain and swelling, areas of localized tenderness, and morning stiffness. Each question is scored on a 0–10 scale, with higher scores indicating greater disease activity. To calculate the BASDAI score, the formula is ((Q1 + Q2 + Q3 + Q4) + ((Q5 + Q6)/2))/5. The BASDAI has been validated for assessing disease activity and is instrumental in monitoring treatment efficacy and progression in axSpA patients [2].

ASDAS: The ASDAS is a composite measure used to assess disease activity in axSpA. It incorporates both clinical variables and acute phase reactants (CRP or ESR) to calculate a score that ranges from low to high/very high disease activity. The formula of CRP-based ASDAS is 0.12 × Back Pain + 0.06 × Duration of Morning Stiffness + 0.11 × Patient Global + 0.07 × Peripheral Pain/Swelling + 0.58 × Ln (CRP+1). The equation for ESR-based ASDAS is 0.08 × Back Pain + 0.07 × Duration of Morning Stiffness + 0.11 × Patient Global + 0.09 × Peripheral Pain/Swelling + 0.29 × √(ESR). ASDAS is designed to be more sensitive to changes in disease activity compared to the BASDAI and provides a quantitative estimate of disease severity [2].

BASFI: The BASFI is a measure of functional impairment in patients with axSpA, focusing on the impact of the disease on daily activities. It consists of 10 questions, each addressing different aspects of physical function, such as dressing, bending, walking, and sitting. The responses are scored from 0 (no difficulty) to 10 (extreme difficulty). The calculation is done by adding the results of the 10 items and dividing them by 10. The BASFI is useful for assessing the functional limitations caused by disease and is often used alongside disease activity indices like BASDAI and ASDAS to gain a comprehensive view of the patient’s health status [2].

ASAS health index: The ASAS HI has been developed under the auspices of the Assessment of Spondyloarthritis International Society (ASAS) to assess health in patients with all forms of spondyloarthritis (SpA) (specifically radiographic and non-radiographic axial SpA as well as peripheral SpA). This self-report questionnaire measures functioning and health across 17 aspects of health and 9 environmental factors (EF) in patients with SpA. It is calculated by summing the scores of 17 items, where each item is scored as 1 if the individual agrees with the statement and 0 if they disagree (range 0–17). The higher the score, the greater the impact of the disease on health. The items measure the concept of ‘functioning, disability and health’—a concept that is conceptualized in the International Classification of Functioning, Disability and Health (ICF). The ICF, a model to systematically classify and describe functioning, disability, and health in human beings, has been used by ASAS as a basis to define a core set of items that are typical and relevant for patients with axSpA [2].

RAPID3: RAPID3 is calculated by summing the scores of three components: physical function, pain, and global health estimate, each scored on a scale of 0–10. The individual scores are added together to obtain a total RAPID3 score, ranging from 0 to 30. This total score can then be divided by 3 to give a 0–10 score for easier comparison with other indices. Disease severity may be classified on the basis of RAPID3 scores: >12 = high; 6.1–12 = moderate; 3.1–6 = low; < or =3 = remission [2].

### 2.4. Clinical Scenarios for Personalized Stratification

To reflect clinical heterogeneity and explore personalized biomarker performance, patients were stratified into several predefined subgroups:

- Disease activity: active (BASDAI ≥ 4 or ASDAS ≥ 2.1) vs. inactive/low activity [2].
- Disease duration: early axSpA (≤2 years) vs. established disease (>2 years) [7].
- Disease impact: high (ASAS-HI > 5) vs. low [8].
- Severity: severe (RAPID3 > 6) vs. non-severe [9].
- Therapy exposure: with vs. without current biologic therapy.

### 2.5. Statistical Analysis

Descriptive statistics were used to summarize the study population. Means (standard deviations) or medians (interquartile ranges) were used for continuous variables, and proportions were used for categorical variables. Between-group comparisons were made using Student’s *t*-test or Mann–Whitney U test for continuous variables and chi-squared or Fisher’s exact test for categorical variables. Pearson correlation coefficients were calculated to assess the relationship among ESR, CRP, and clinical outcomes. To identify the optimal biomarker thresholds discriminating between clinical states (e.g., high vs. low disease activity), the Youden index was applied. The discriminatory capacity of ESR and CRP was assessed via receiver operating characteristic (ROC) curves, and the area under the curve (AUROC) was calculated. A *p*-value < 0.05 was considered statistically significant. All analyses were performed using R software (version 4.3.1, “Beagle Scouts”, New Zealand).

## 3. Results

### 3.1. Summary of Study Population

A total of 127 (38.5%) women and 203 (61.5%) men were included, mean age of 47.6 (SD 12.9) years and median disease duration of 8 [IQR 4–16] years. At inclusion, 209 (63.3%) patients were receiving biological therapies, mostly anti-TNFα. Most patients had radiographic axSpA (80%) with a 3:1 ratio favoring men. Table 1 summarizes the characteristics of the study population as well as sex-based differences.

### 3.2. Differences Based on HLA-B27

At inclusion, individuals positive for HLA-B27 were significantly younger than their negative counterparts (mean age: 46.3 ± 13.1 vs. 49.9 ± 11.8 years; *p* = 0.016) and had a longer disease course (median duration: 9.5 [IQR 4.0–19.3] vs. 6.0 [IQR 3.0–11.0] years; *p* = 0.003). NSAID usage was more common in HLA-B27-positive patients (75.3%) compared to those without the allele (61.7%; *p* = 0.02). With respect to extra-articular manifestations, uveitis was notably more frequent in the HLA-B27-positive group (19.4% vs. 6.4%; *p* = 0.006), whereas inflammatory bowel disease was more prevalent in HLA-B27-negative individuals (21.3% vs. 5.3%; *p* < 0.001). Disease activity scores were also higher in the HLA-B27-negative cohort, as reflected by BASDAI (4.26 ± 2.42 vs. 3.45 ± 2.39; *p* = 0.006) and ASDAS (2.21 ± 0.81 vs. 2.03 ± 0.87; *p* = 0.035). In terms of cardiometabolic comorbidities, diabetes was slightly less common among HLA-B27-positive patients (2.6% vs. 7.4%; *p* = 0.06). Bilateral sacroiliitis was observed more frequently in HLA-B27-positive subjects (92.0% vs. 74.5%; *p* < 0.001). No significant differences in ESR or CRP levels were found between the two groups.

### 3.3. Correlation Between Inflammatory Markers and Outcome Measures

**Figure 1:**
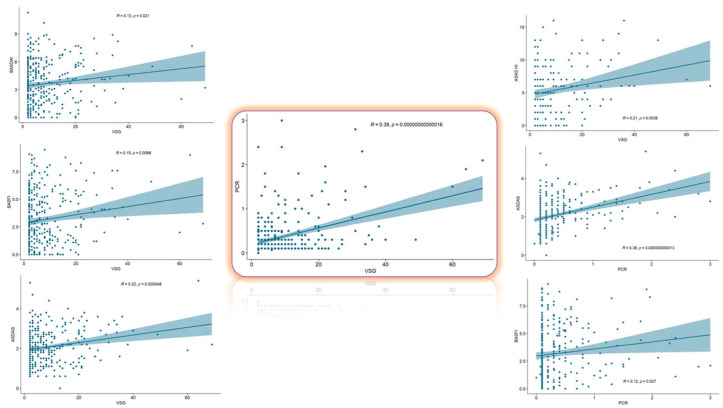
Correlations between ESR and CRP with the different study outcome measures. In general, weak positive correlations were observed between both biomarkers and the different outcome measures. The erythrocyte sedimentation rate correlated with a greater number of outcome measures than C-reactive protein. See text for details. VSG = ESR (erythrocyte sedimentation rate); PCR = CRP (C-reactive protein); BASDAI: Bath Ankylosing Spondylitis Disease Activity Index; ASDAS: Ankylosing Spondylitis Disease Activity Score; BASFI: Bath Ankylosing Spondylitis Functional Index; ASAS HI: Assessment of Spondyloarthritis International Society Health Index.

ESR and CRP levels were moderately correlated (r = 0.39, *p* < 0.001). ESR showed weak but significant correlations with BASDAI (r = 0.13, *p* = 0.021), ASDAS (r = 0.22, *p* < 0.001), BASFI (r = 0.15, *p* = 0.007), and ASAS HI (r = 0.21, *p* = 0.004), but not with RAPID3 or mSASSS. CRP was correlated with ASDAS (r = 0.36, *p* < 0.001) and BASFI (r = 0.12, *p* = 0.027) and showed a borderline correlation with mSASSS (r = 0.16, *p* = 0.06). No significant associations were observed between CRP and BASDAI, ASAS HI, or RAPID3. ESR appeared to reflect a broader spectrum of patient-reported outcomes. Correlation plots are shown in Figure 1.

### 3.4. Biomarker Discrimination of Clinical Scenarios

Neither ESR nor CRP demonstrated adequate discriminative power (AUROC < 0.70) for active disease (BASDAI ≥ 4 or ASDAS ≥ 2.1), high impact (ASAS HI > 5), or high severity (RAPID3 > 6). The best-performing cut-off was a CRP ≥ 0.15 mg/dL for very high disease activity (ASDAS > 3.5), with an AUROC of 0.68.

### 3.5. Stratification by Disease Duration

Among 299 patients with available data, 45 had early axSpA (≤2 years). In this subgroup, CRP ≥ 0.56 mg/dL demonstrated moderate discriminative capacity for very high disease activity (ASDAS > 3.5), with an AUROC of 0.74 (95% CI: 0.43–1.00). No discriminant thresholds were identified for either biomarker in established disease. These findings highlight the potential role of CRP in early disease monitoring.

### 3.6. Discriminatory Thresholds of ESR and CRP Based on Biologic Drug Exposure

Finally, we looked for discriminative cut-offs for ESR and CRP based on whether or not patients were on biological therapies. An ESR value ≥ 8.5 mm/h was highly discriminatory for very high disease activity according to ASDAS in patients without exposure to biologics, with an AUROC of 0.86 (95% CI: 0.70–1). On the other hand, a CRP of 1.88 mg/dL was highly discriminatory for an ASDAS > 3.5 in patients without biological therapy, with an AUROC of 0.84 (95% CI: 0.54–1). In patients undergoing treatment with biologics, no ESR or CRP value achieved discriminatory capacity in any of the scenarios analyzed.

**Figure 2:**
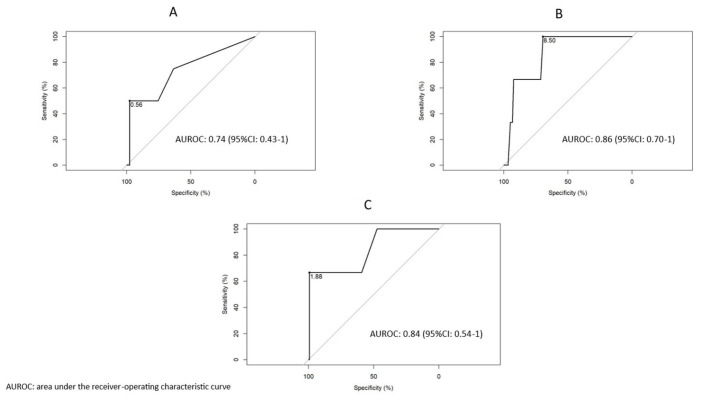
Area under the receiver-operating characteristic curve to discriminate the very high disease activity level according to the ASDAS threshold. (**A**) The best value according to the Youden index to discriminate an ASDAS > 3.5 (very high disease activity) was a CRP ≥ 0.56 mg/dL in patients with early disease (≤2 years). (**B**) An ESR value ≥ 8.5 mm/h was highly discriminatory for an ASDAS > 3.5 in patients without exposure to biologics. (**C**) In patients not exposed to biologic therapy, a CRP ≥ 1.88 discriminated with high accuracy an ASDAS > 3.5. AUROC: Area under the receiver-operating characteristic curve; ASDAS: Ankylosing Spondylitis Disease Activity Score; CRP. C-reactive protein; ESR: erythrocyte sedimentation rate.

Figure 2 illustrates discriminatory thresholds by disease duration and biologic drug exposure.

### 3.7. ESR in Axial Spondyloarthritis: Highly Specific in Different Clinical Scenarios

An ESR ≥ 13.5 mm/h had a high specificity for discriminating active BASDAI (0.86). The same value was also highly specific (0.85) for a high disease impact according to the ASAS HI. In patients with early-onset disease, an ESR ≥ 9 mm/h discriminated with high specificity (0.89), but moderate sensitivity (0.53) was noted for a BASDAI ≥ 4. An ESR value ≥ 21.5 mm/h was highly specific (0.88) but had low sensitivity (0.50) for very high disease activity according to ASDAS. An ESR cutoff ≥ 13.5 mm/h was highly specific (0.87) for high-impact disease according to the ASAS HI in established disease. Among patients without biological therapy, an ESR ≥ 13.5 mm/h was highly specific for both active BASDAI (0.87) and high ASDAS (0.86). In patients undergoing biological treatment and with high-impact ASAS HI, an ESR ≥ 21.5 mm had a specificity close to 100% (0.98).

### 3.8. CRP in Axial Spondyloarthritis: Highly Specific in Different Clinical Scenarios

A CRP value ≥ 0.38 mg/dL was specific (0.82) for high disease activity according to the ASDAS, but with low sensitivity (0.43). For a high-severity RAPID3, a CRP of 0.43 mg/dL was highly specific (0.91), but with low sensitivity (0.32). A CRP ≥ 0.38 mg/dL was specific (0.82) for high ASDAS activity in patients with established disease, while a CRP ≥ 0.56 mg/dL had a specificity close to 100% (0.97) for the very high ASDAS activity category. Also, in patients with established disease, a CRP value ≥ 0.43 mg/dL was highly specific for RAPID3 > 6. A CRP ≥ 0.45 mg/dL was highly specific for high BASDAI (0.89) and high ASDAS (0.94) in patients without biological therapy. Finally, CRP values ≥ 0.34 and ≥ 0.45 were specific for a high-severity RAPID3, in patients with (0.88) and without (0.95) biologics, respectively.

**Figure 3:**
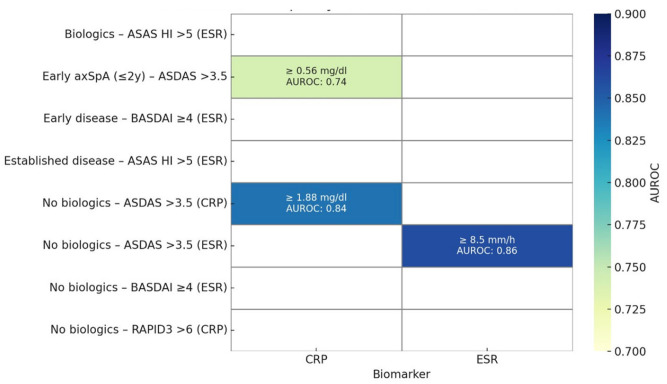
Discriminative capacity of ESR and CRP across clinical scenarios in axial spondyloarthritis. This heatmap illustrates the performance of erythrocyte sedimentation rate (ESR) and C-reactive protein (CRP) in distinguishing high disease activity states across selected clinical scenarios in axial spondyloarthritis (axSpA). Each cell displays the biomarker cut-off with the corresponding area under the receiver operating characteristic curve (AUROC). The highest discriminatory capacities were observed for ESR ≥ 8.5 mm/h and CRP ≥ 1.88 mg/dL in patients not exposed to biologic therapy and for CRP ≥ 0.56 mg/dL in early axSpA. These results highlight the potential utility of tailored biomarker interpretation depending on disease stage and treatment status.

Figure 3 summarizes discriminative capacity of ESR and CRP across clinical scenarios in axSpA.

## 4. Discussion

In this study, we evaluated the performance of erythrocyte sedimentation rate (ESR) and C-reactive protein (CRP) in a large, well-characterized cohort of patients with axial spondyloarthritis (axSpA), focusing on their utility across diverse clinical scenarios. Our findings reveal that while both biomarkers display limited overall sensitivity for active disease, they demonstrate high specificity in certain patient subgroups and exhibit distinct performance profiles depending on disease stage and therapeutic exposure. These results support a more personalized approach to biomarker interpretation in axSpA.

ESR and CRP continue to be the cornerstone inflammatory biomarkers employed in the monitoring of axSpA [2]. The presence of elevated CRP is considered a supportive feature in current axSpA classification criteria [6]. Both indicators are integral to the ASDAS index, a key tool for assessing disease activity [2,4,5]. Nevertheless, their diagnostic utility is constrained by limited sensitivity and specificity, as only 40–50% of patients with active disease exhibit elevated levels [4,5]. Meanwhile, in those with early axSpA, CRP and ESR did not differ from that of chronic low back pain patients [10,11]. However, CRP has been associated with the possibility of a better response to biological therapy, structural damage, and its progression, and it also shows good sensitivity to change after the initiation of advanced therapies. Thus, today, CRP (and perhaps somewhat less ESR) remains the standard biomarker for disease assessment, both in clinical trials and routine practice [2,4,5,12]. Lastly, CRP-ASDAS has been agreed upon as the activity index to be used in clinical trials and daily practice to monitor the disease and select patients who are candidates for high-impact therapies [3].

While both ESR and CRP reflect systemic inflammation, CRP appears to be more closely associated with clinical disease activity in axSpA, particularly during active disease states. This is likely due to CRP’s sensitivity to IL-6-mediated inflammation, which is more directly tied to the immune mechanisms involved in axSpA pathogenesis [1,2,4,5]. In contrast, ESR’s correlation with disease activity might reflect a broader, less specific inflammatory state. Additionally, CRP’s correlation with treatment response, particularly to biologics like TNF inhibitors, may indicate that it is a more dynamic marker that responds promptly to therapeutic interventions, unlike ESR, which may show slower changes [4,5]. In conclusion, while both ESR and CRP serve as valuable biomarkers in monitoring axSpA, they each provide distinct insights. ESR offers a broader perspective on the disease process, possibly reflecting a more generalized inflammatory burden. CRP, on the other hand, is more specifically linked to the active inflammatory processes of axSpA and might be more responsive to treatment changes, especially in biologic-naïve patients [2,4,5]. This distinction underscores the importance of a personalized biomarker strategy in managing axSpA, where both markers can complement each other depending on the disease stage and therapeutic context.

That said, do we really have information that allows us to ensure the superiority of one of these biomarkers over the other? Certainly, ESR seems to be a nonspecific measure of inflammation that may be influenced by a variety of other nonrheumatic conditions and comorbidities; while, on the other side, CRP seems to outperform ESR in assessing different aspects of axSpA as mentioned above [2,4,5]. However, some studies reported that high ESR is also independently associated with structural disease progression in patients with nr-axSpA [5]. Here, we were unable to detect a positive correlation between ESR levels and structural damage (with CRP this correlation only bordered on statistical significance), but we did find that ESR values in general were positively correlated with a higher number of outcome measures (activity, function, severity and impact) compared to CRP, which was only significantly correlated with activity and function. On the other hand, although the majority of patients fell into the r-axSpA category, we did not detect significant differences between patients with and without radiographic forms of the disease in terms of CRP or ESR values. Moreover, while both ESR and CRP were incorporated into their respective composite indices (ASDAS-ESR and ASDAS-CRP), the predictive value of these biomarkers remained modest overall, with ESR showing broader associations with clinical outcomes. Thus, it appears that ESR more comprehensively captures the disease process in axSpA.

On the other hand, when analyzing the discriminatory ability of different thresholds of both biomarkers in the different clinical scenarios proposed, we were unable to demonstrate any superiority of one over the other. In fact, the only discriminant values were found among patients included under the ASDAS very high activity label. Thus, for example, in patients with recent-onset disease, a CRP close to 0.6 mg/dL was discriminant for a very high ASDAS activity (AUROC 0.74). Likewise, in patients not exposed to biological therapies, an ESR ≥ 8.5 mm/h (AUROC 0.86) and a CRP ≥ 1.88 mg/dL (AUROC 0.84) indistinctly discriminated the ASDAS very high activity state. Finally, both inflammatory markers were highly specific when tested in some of the different clinical situations, with neither of them standing out over the other in that regard.

Our results are consistent with those of other studies, in that both biomarkers largely lack sensitivity for detecting active disease, particularly in early stages or in patients with relatively low inflammatory burden [4,5,10]. This can lead to false negatives if the interpretation of disease activity is limited to the assessment of these two biomarkers and other aspects (e.g., imaging) are not considered. In contrast, the sensitivity and specificity of both markers appear to improve only in patients with the highest ASDAS scores, which limits their practical usefulness to a relatively limited group of patients. This inconsistency with the two classic inflammatory biomarkers in axSpA has led to an intense effort to find and validate new biomarkers in this entity. Numerous emerging biomarkers have shown potential associations with the diagnosis and disease activity of axSpA in various studies. These include BMP7, CCL11, anti-CD74 antibodies, circulating microRNAs, the CRP-to-albumin ratio (CAR), fibrinogen-to-albumin ratio (FAR), ERAP1, EEF1E1, H-ficolin, L-ficolin, LINC00311, pentraxin 3, serum calprotectin, and tenascin-C. Furthermore, markers such as CD163, CD206, anti-TNF-α neutralizing antibodies, lipocalin 2, oncostatin M, neutrophil extracellular traps (NETs), semaphorin 3A, and serum calprotectin have demonstrated value in evaluating therapeutic response, thereby contributing to the optimization of biologic treatment strategies. Given the elevated cardiovascular risk in axSpA, additional biomarkers—such as CRP, complement C3, omentin-1, osteoprotegerin, sclerostin, serum calprotectin, vaspin, and visfatin—have been proposed for early detection and potential improvement of cardiovascular outcomes in this population [4]. However, despite growing interest, the clinical application of many of these novel biomarkers remains limited, largely due to the absence of robust external validation across independent cohorts.

C-reactive protein could play a role in monitoring the disease in its earliest stages, when proinflammatory mechanisms have not yet been effectively counteracted by available therapies for axSpA. In fact, it has been shown that CRP levels correlate with inflammatory activity measured by MRI and that changes in CRP levels accompany the changes detected on MRI. This is especially true for changes in the spine, and less so for changes detected in the sacroiliac joints. This may partly explain the relatively low sensitivity of this biomarker for detecting the disease (which in most cases is based on imaging findings in the sacroiliac joints) [4,5,10,11].

Some aspects of our study may have a translation into practice. Thus, “subinflammatory” values of both markers performed equally well for classification purposes, both from the point of view of the activity and severity as well as the impact that the disease generates in different real-life clinical situations in axSpA. Considering that both ESR and CRP are commonly requested markers for disease monitoring, our findings could have a place in the informed decision-making scenario in routine care for these patients [13]. In fact, very recently a proposal has been made to lower the discriminatory thresholds for active disease in both psoriatic arthritis and axSpA [14,15]. Logically, these thresholds need further endorsement.

These findings underscore the value of a personalized biomarker strategy—where ESR and CRP are not used interchangeably, but selectively interpreted based on disease stage, therapeutic context, and clinical phenotype. High-specificity thresholds may be particularly useful in identifying patients with substantial inflammatory activity who may benefit from therapeutic escalation, especially when symptom-based indices are inconclusive.

Among the weaknesses of this study, we must highlight the retrospective nature of the data collection, which surely introduces biases and confounding factors that contribute to reducing the quality of this information. On the other hand, patients included under the ASDAS label of very high activity represented a minority of cases. Finally, we have to assume that both biomarkers may experience changes unrelated to the inflammatory activity of the disease. By including a substantial number of cases, the different outcomes analyzed, as well as the different scenarios related to the use of biologics or the duration of the disease, we have contributed to further clarify the role of both markers in axSpA monitoring.

## 5. Conclusions

In this real-world study of axSpA, neither ESR nor CRP demonstrated robust discriminative power across the full spectrum of disease activity, severity, or impact. However, their performance varied significantly depending on disease duration and treatment exposure. ESR showed broader associations with clinical outcomes and may better capture the overall disease burden, particularly in early or untreated patients. CRP remained more closely tied to inflammatory activity but was less consistently associated with patient-reported impact. These findings support a personalized approach to biomarker use in axSpA, wherein ESR and CRP are interpreted within the context of patient-specific variables such as disease stage and therapeutic status. A dual-marker strategy may enhance disease stratification and help guide individualized treatment decisions in routine care. However, neither CRP nor ESR can be used as definitive markers for a personalized diagnosis of axSpA. These biomarkers are not sufficiently specific or sensitive, and future research should focus on identifying new biomarkers that can provide a more accurate and individualized assessment of disease activity and severity.

## Figures and Tables

**Table 1:** Characteristics of the study population and differences by sex.

	Total N: 330	Women N: 127	Men N: 203	*p*-Valor
Age, years, mean (SD)	47.8 (12.9)	47.3 (13.0)	48.1 (12.9)	NS
Dis. duration, years, median [Q1, Q3]	8.0 [4.0–16.0]	6.0 [3.0–12.0]	10.0 [5.0–20.0]	<0.001
Education, n (%)				
Primary	87 (26.4)	38 (29.9)	49 (24.1)	NS
Secondary	157 (47.6)	52 (40.9)	105 (51.7)	NS
University	86 (26.0)	37 (29.2)	49 (24.2)	NS
Associated conditions, n (%)				
Enthesitis	26 (7.9)	7 (5.5)	19 (9.4)	NS
Uveitis	52 (15.8)	19 (15.0)	33 (16.3)	NS
IBD	34 (10.3)	16 (12.6)	18 (8.9)	NS
Comorbidities, n (%)				
Diabetes	15 (4.5)	5 (3.9)	10 (4.9)	NS
HBP	47 (14.2)	14 (11.0)	33 (16.3)	NS
Obesity	39 (11.8)	16 (12.6)	23 (11.3)	NS
Smoking	115 (34.8)	41 (32.3)	74 (36.5)	NS
Therapy, n (%)				
NSAIDs	233 (70.6)	94 (74.0)	139 (68.5)	NS
Biologics	209 (63.3)	77 (60.6)	132 (65.0)	NS
csDMARDs	57 (17.3)	22 (17.5)	35 (17.2)	NS
Laboratory parameters				
CRP (mg/dL), median [Q1, Q3]	0.20 [0.10–0.40]	0.20 [0.10–0.50]	0.10 [0.10–0.40]	NS
ESR (mm/h), median [Q1, Q3]	5.0 [2.0–10.8]	8.0 [3.0–17.0]	4.0 [2.0–8.0]	<0.001
HLA-B27, n (%)	233 (70.6)	80 (63.0)	147 (72.4)	0.07
Structural damage, n (%)				
r-axSpA	264 (80)	62 (48.8)	202 (99.5)	<0.001
nr-axSpA	66 (20)	65 (51.2)	1 (0.5)	<0.001
High-grade sacroiliitis	287 (87.0)	98 (77.2)	189 (93.1)	<0.001
Syndesmophytes	93 (28.2)	18 (14.2)	75 (36.9)	<0.001
mSASSS, median [Q1, Q3]	6.0 [2.0–15.0]	4.0 [0.0–8.0]	8.0 [4.0–20.0]	<0.001
Treatment				
Anti-TNFα	167 (50.6)	67 (52.7)	100 (49.3)	NS
Anti-IL17	42 (12.7)	15 (11.8)	27 (13.3)	NS
Anti-JAK	22 (6.7)	8 (6.3)	14 (6.9)	NS
Outcome measures				
BASDAI, mean (SD)	3.6 (2.4)	4.4 (2.4)	3.2 (2.3)	<0.001
ASDAS, mean (SD)	2.1 (0.8)	2.3 (0.8)	1.9 (0.8)	<0.001
BASFI, mean (SD)	3.2 (2.4)	3.5 (2.4)	2.9 (2.4)	0.031
ASAS HI, mean (SD)	5.4 (4.0)	6.1 (3.9)	4.9 (3.9)	0.026
RAPID3, mean (SD)	9.4 (6.6)	11.5 (6.2)	8.2 (6.6)	0.005
BASDAI_categories, n (%)				
≥4	146 (44.2)	73 (57.5)	73 (36.0)	<0.001
ASDAS_categories, n (%)				
≥2.1	147 (44.7)	71 (55.9)	76 (37.6)	0.002
ASAS-HI, n: 200 (%)				
>5	94 (47)	46/79 (58.2)	48/121 (39.7)	0.012
RAPID3, n: 131 (%)				
>6	87 (66.4)	40/49 (81.6)	47/82 (57.3)	0.008

N, n: number; SD: standard deviation; Dis: disease; Q1: first quartile; Q3: third quartile; IBD: inflammatory bowel disease; HBP: high blood pressure; NSAIDs: non-steroidal anti-inflammatory drugs; csDMARDs: conventional synthetic disease-modifying antirheumatic drugs; CRP: C-reactive protein; ESR: erythrocyte sedimentation rate; HLA: human leukocyte antigen; r-axSpA: radiographic axial spondyloarthritis; nr-axSpA: nonradiographic axial spondyloarthritis; mSASSS: modified Stoke ankylosing spondylitis spinal score; BASDAI: Bath Ankylosing Spondylitis Disease Activity Index; ASDAS: Ankylosing Spondylitis Disease Activity Score; BASFI: Bath Ankylosing Spondylitis Functional Index; ASAS HI: Assessment of Spondyloarthritis International Society Health Index; RAPID3: routine assessment of patient index data 3.

## Data Availability

The materials and raw data described in the manuscript will be freely available to any researcher without breaching any participant’s confidentiality. To facilitate the revision of the results by other researchers, a file with the patient data is available as an excel file upon request to the corresponding author.
